# Dietary Quality Indices and Biochemical Parameters Among Patients With Non Alcoholic Fatty Liver Disease (NAFLD)

**DOI:** 10.5812/hepatmon.10943

**Published:** 2013-07-13

**Authors:** Ali Hashemi kani, Seyed Moayed Alavian, Ahmad Esmaillzadeh, Peyman Adibi, Leila Azadbakht

**Affiliations:** 1 Food Security Research Center, Isfahan University of Medical Sciences, Isfahan, IR Iran; 2 Department of Community Nutrition, School of Nutrition and Food Science, Isfahan University of Medical Sciences, Isfahan, IR Iran; 3 Baqiyatallah Research Center for Gastroenterology and Liver Disease, Baqiyatallah University of Medical Sciences, Tehran, IR Iran; 4 Integrative Functional Gastroenterology Research Center, Isfahan University of Medical Sciences, Isfahan, IR Iran

**Keywords:** Diet Therapy, Healthy People Programs, Biochemical Processes, Non-alcoholic Fatty Liver Disease

## Abstract

**Background:**

Dietary intake might have important role in non-alcoholic fatty liver diseases (NAFLD). Although, there are some reports on dietary intake and anthropometrics measurements, few studies have focused on the markers of assessing whole diet like dietary quality indices.

**Objectives:**

Therefore, our aim was to determine the diet quality indices and biochemical parameters among patients with NAFLD and healthy individuals.

**Patients and Methods:**

This case-control study was performed on 100 patients with NAFLD and 100 healthy subjects who were attending to Gastrointestinal Research Center, Baqiyatallah University, Tehran, Iran during the recent years. Usual dietary intake was assessed by three dietary records (one weekend and two week days). Healthy eating index (HEI), dietary diversity score (DDS), dietary energy density (DED), mean adequacy ratio of nutrients (MAR) were assessed according to the standard methods.

**Results:**

Patients with NAFLD had higher body mass index, weight and waist circumference compared to the healthy group (P < 0.05). Serum levels of liver enzymes, triglyceride, LDL, BUN, and uric acid were higher in patients with NAFLD (P < 0.05). Although patients with NAFLD had higher energy, carbohydrate and fat intake, their values for antioxidant vitamins, calcium and vitamin D were lower than healthy subjects (P < 0.05). HEI and MAR were higher among healthy group, and DED was lower among them. Nutrient adequacy ratio for calcium, vitamin D, and antioxidant micronutrients were lower in patients with NAFLD (P < 0.05).

**Conclusions:**

It seems that dietary quality indices may be associated with NAFLD. Calcium, vitamin D, and antioxidant micronutrients intake might be lower among patients with NAFLD based on this case-control study. Further prospective studies should be conducted in this regard.

## 1. Background

Nonalcoholic Fatty liver disease (NAFLD) includes a wide range of clinical signs of liver damage which can progress to cirrhosis and liver failure (1). This disease shows the hepatic manifestation of metabolic syndrome; therefore, can increase the risk of cardiovascular diseases (2). Evidences have shown that NAFLD affects 20 to 30 percent of adults in western countries (3), and 12 to 24 percent of general population in Asians (4). In Iran the mortality rate of 1% in subjects more than 15 years is related to this disease (5). It is reported to be around 20% in Golestan province in Iran (6). Previous epidemiological studies among Iranians showed that ALT, total cholesterol, and waist circumference are the independent predictors of NAFLD (7). In another study, NAFLD was related to male gender, overweight, diabetes mellitus, and living in urban areas (8). Furthermore, Bahrami et al. (9) showed that 87% of patients with NAFLD were obese or overweight, 75.5% were dyslipidemic, and 80% were diabetic. A complex interaction between genetic and environmental factors is responsible for NAFLD etiology (10). Among environmental factors dietary intake is an important issue (11). NAFLD is mostly common among obese or overweight subjects and also among those with the presence of the features of insulin resistance syndrome (12). Diet is associated with obesity as well as all of these mentioned abnormalities. Excessive amount of refined carbohydrate (13, 14) as well as high amount of fat intake (15, 16) is responsible for this disease based on some evidence worldwide. Furthermore, different kinds of fat consumption are also related to NAFLD (17). A recent study in China showed that frequent consumption of desserts and salty foods is related to NAFLD. They showed that Intake of coarse cereals, potatoes, vegetables, fruits, and milk was significantly lower, and intake of red meat, viscera, candies and pastries, cooking oil, and total energy was significantly higher in participants with NAFLD than in controls (18). Another recent investigation among children showed that high dietary intakes of CHO and SFA and a low dietary consumption of fiber and n-3 FA were positively associated with the pathogenesis of NAFLD (19). Dietary cholesterol intake was also related to this disease (20). A recent study showed no significant association between carbohydrate quality and liver enzymes (21). Most of the reported dietary determinants of NAFLD are not from the Middle East. However, there is a special pattern of obesity in the Middle East which can be called as the Middle Eastern pattern of obesity (22) which shows the necessity of doing epidemiologic studies in the field of dietary intake and NAFLD separately in this region (22). Especially the central adiposity is prevalent among Asian population which may be related to pathology of NAFLD in this region (23, 24).

Fatty liver is associated with elevated serum ALT and AST and GGT concentration (25). However, 10% to 15% of general population with fatty liver may show normal levels of plasma liver enzymes (26). It is interesting to have data regarding the level of enzymes in patients with NAFLD in different regions of the world and to see the fluctuations of these enzymes in different epidemiological studies (26). A recent study showed that serum levels of lipid profiles are also related to NAFLD (20). Furthermore, assessing the association between lipid profiles, glycemic indices and blood pressure and NAFLD is important to be clarified. Recently there are some reports of the link between cardiovascular risks and kidney disorders among patients with NAFLD (26). So, it is important to assess the biochemical indices of patients with NAFLD in different regions of the world. Although there are some reports regarding the association between dietary intake and NAFLD, but they just assessed some components of a diet like some macro or micro nutrients or some kinds of foods (15, 17, 20, 21). However, there are no reports regarding the association of whole diet intake and NAFLD. As it is not important to just consider some nutrients or foods and it is important to evaluate the whole of diet, we should determine the association of the whole diet with NAFLD by using indices that show the whole characteristics of the diet. Usually, most nutrients are consumed along with each other in a diet, so they may have synergistic effects on each other. Therefore, it is logical to assess the diet that involves the entire nutrients and foods intake. Diet quality indices such as healthy eating index (HEI), dietary diversity score (DDS), mean adequacy ratio of nutrients (MAR), and dietary energy density (DED) are the indices which show the whole diet characteristics among Iranian population. We hypothesized that those subjects with higher score of HEI might follow a more healthier diet which might be associated with higher fruit and vegetable and dietary photochemicals, and therefore might have probably an inverse association with NAFLD. We thought that those subjects with lower DDS might have lower antioxidant intake which might be related to NAFLD, because previous studies in Iran showed that higher DDS were associated with healthier dietary intake. We assumed that those with higher scores of MAR might consume more micronutrients which might be correlated with lower prevalence of NAFLD. Furthermore, higher DED might positively correlate with NAFLD.

## 2. Objectives

Therefore, our aim was to evaluate dietary intake, dietary quality indices, as well as anthropometric and biochemical indices among patients with NAFLD and healthy group.

## 3. Patients and Methods

### 3.1. Subjects

This case-control study was performed on 100 patients with NAFLD and 100 healthy subjects who were attending to Baqiyatallah Research Center for Gastroenterology and Liver Disease, Baqiyatallah University, Tehran, Iran during the recent years. We determined the number of case and control according to this formula: n = [(R+1)/R] × {δ2 (Zβ+Zα/2)}/d2. R was considered as 1/1. δ was considered as 3 according to the previous studies (21). d was considered as 1.25. Therefore we need 93 patients in each group. Patients with NAFLD were those with increased level of ALT and AST (ALT and AST more than 30 in men, and more than 20 in women), and NAFLD was confirmed by sonography. Healthy subjects were carriers of hepatitis B who were found to have positive results accidently in a blood sampling test, and they were attending to Baqiyatallah Research Center for Gastroenterology and Liver Disease for starting the treatment as they thought that they had hepatitis B. However, in further evaluation, it would be confirmed that they were not active patients and they did not need any treatment and they were just carriers of hepatitis B. So, the reason for attending in research center for control group was that they have been recognized as the positive cases of hepatitis B accidentally following a biochemical blood test and they were attending in research center to start the treatment. However, after attending they were recognized just as a carrier and they were requested to refer again every six months. We randomly chose the control group among the carriers of hepatitis B who were recently attended in the research center. Random selection was performed based on a computer program. Control group was selected as the same time of the tested. However, the case group was chosen according to the inclusion criteria (having NAFLD which was confirmed by sonography and increasing level of ALT and AST). Case group patients were new cases and they were not treated before the study. We used the documents which were recorded in the research center. Telephone number, sex, age and liver enzyme status were used from the documents. However, we checked the liver enzymes again after they came to the lab. Before entering the study to omit other possible reasons for increased levels of ALT, and AST, some tests were performed. Serum ferritine, TIBC, and plasma iron level were measured for detecting hemocromatosis. Other tests for detecting viral hepatitis, Wilson’s disease, autoimmune disorders of the liver were also conducted. 

Then for completing data on dietary intake and other information we called the patients to invite them to the research center in a special date. Participants with a history of certain diseases (diabetes, cardiovascular disease, myocardial infarction or stroke and cancer) were excluded. We also excluded pregnant and lactating women and subjects who had arbitrary special diet. Other information was also gathered after they attended in the research center. This study was as interview administered and it was not self-administered. There was an interviewer in the present study and the interviewer was responsible for the unresponded questions. So, all the patients responded completely to the survey questions. This study was approved by the research council and ethics committee of Food Security Research Center and School of Nutrition and Food Science, Isfahan University of Medical Sciences, Isfahan, Iran (Research project number: 185190). 

### 3.2. Assessment of Dietary Intake

Usual dietary intake was assessed by three dietary records (one weekend and two week days) (27). By using household measures the amount of each food item was converted to gram per day. Food records were analyzed using the NUTRITIONIST IV. Dietary quality indices were measured by the nutritionist in this project. According to previous studies (28-31) we considered those with energy intake lower than 800 kcal per day as under reporters, and those with energy intake higher than 4200 kcal per day as over reporters. Dietary Diversity Score: Dietary diversity score was calculated according to Kant et al. (32, 33) method. Five food groups based on the food groups introduced by the USDA`S Food Guide Pyramid (bread-grains, vegetables, fruits, meats and dairy) were considered (34). The main mentioned food groups were divided into 23 subgroups. These subgroups show the dietary diversity across the groups of the Food Guide Pyramid (34). Seven subgroups were considered for bread-grain group (refined bread, biscuits, macaroni, whole bread, corn flakes, rice, and refined flour). We divided fruits into two subgroups (fruit and fruit juice, berries and citrus). Vegetable was divided into seven subgroups (vegetables, potato, tomato, other starchy vegetables, legumes, yellow vegetables, green vegetables). Four subgroups were considered for meat (red meat, poultry, fish, and eggs). Three subgroups (milk, yogurt, cheese) were considered for dairy group. For determining the consumers of each group we considered consumption of one-half of serving for each group. Each of the five food groups received a maximum diversity score of two of the 10 (5×2 = 10). Total score was the sum of the scores of each of the five main groups. Therefore, the maximum and minimum scores of total dietary diversity were between 0 and 10. The same method was used for calculating each food group diversity score. So, the maximum and minimum scores of food group diversity were 0-2 within each of the food groups. Consuming at least one-half serving of any subgroups on one day according to the Food Pyramid quantity criteria was enough for considering some one as the consumer of each food group. For example in bread-grain group, if a person was the consumer of whole grain, macaroni, and biscuits his or her score was calculated as (3÷7) × 2 = 0.85. Therefore, the diversity score of the bread grain group was 0.85.

### 3.3. Healthy Eating Index (HEI)

The method introduced by Kennedy et al. ( 35 ) was used for calculating HEI. This index contains 10 different components. The amount of five groups of grains, vegetables, fruits, milk, and meat groups as expressed in servings/day was considered as the first five components of HEI. 10 points were given to the intakes at or recommended amounts for each food group. If anybody consumed no servings of a food group received a score of 0. Between 0 and 10, the scores were calculated proportionately. The percent consumed of total fat and saturated fatty acids, were the 6^th^ and 7^th^ scores, respectively. The score of cholesterol intake and dietary variety was considered as the 8^th^ and 9^th^ components. A full score of 10 points was awarded for diets with < 30% energy from fat, < 10% energy from saturated fat, and < 300 mg cholesterol. The number 10 was related to sodium. A full score of 10 was awarded to those not adding the table salt according to their report. Mean adequacy Ratio and Nutrient adequacy ratios: The ratio of daily individual intakes to standard recommended amounts for subject’s sex and age category was used for calculating nutrient adequacy ratio (NAR). The standard recommended amounts were based on the RDA (Recommended Dietary Allowances) and the DRI (Dietary Reference intake) ( 36 ). The NAR for ten nutrients including vitamin D, vitamin B12, vitamin B6, vitamin A, vitamin B2, niacin, vitamin C, calcium, iron, and zinc were estimated according to the mentioned formula. Mean adequacy ratio (MAR) was calculated as the sum of NARs divided on the number of nutrients (n = 10) ( 37 ). Dietary Energy density: The method of Ledikwe et al. ( 38 , 39 ) was used to determine the ED of the diets. This method calculates ED from food only as energy (kcal)/weight of food (g) excluding nonenergetic beverages. Total energy intake was calculated by summing up energy intakes from all foods. The under reporters and over reporters of energy intake were excluded in this study both in case and control groups. Calorie intake was the determining factor for under reporting and over reporting of energy intake. Table 1 shows the summary table of indices. 

### 3.4. Anthropometric Assessment

Weight and height were measured according to the standard method.

Body mass index (BMI) was calculated by dividing weight (in kilograms) by height squared (in meters squared) (28, 29). 

### 3.5. Evaluation of Blood Pressure

Before the measurement, consumption of tea, coffee, physical activity and a full bladder was questioned, and subjects were requested to rest for 15 min before the measurement. Blood pressure was measured twice by an expert person by using a standard mercury sphygmomanometer. The mean of two measurements was considered as the participant’s blood pressure (31).

### 3.6. Assessment of Biochemical Evaluation

To measure blood glucose levels and serum lipids (including total cholesterol, serum triglycerides and HDL cholesterol), venous blood samples were collected after 10-12 h of fasting. Blood samples were centrifuged for 10 min at 500_g and at 4 °C within 30–45 min of collection. Levels of total cholesterol and triglycerides were determined by using commercial kits (Pars Azmoon, Tehran, Iran). HDL and LDL cholesterol levels were also measured by enzymatic kits (Pars Azmoon, Tehran, Iran). Other biochemical parameters were assessed by commercial kits (31).

### 3.7. Assessment of Other Variables

Information about other variables such as age, demographic data, medical history and medication use was obtained by questionnaires. 

### 3.8. Statistical Analysis

The Statistical Package for Social Science (version 16; SPSS Inc, Chicago IL) was used for all statistical analyses. The normality of variables was checked by Kolmogorov-Smirnov and histogram tests. For comparing the mean of diet quality indices such as HEI, MAR and NAR of 10 nutrients as well as the general characteristics and biochemical parameter among case and control group, independent sample T test was used. As the BMI of two groups were different we adjusted the effect of BMI in a separated model when we wanted to compare the energy intake between the two groups. As difference in BMI would be effective in difference in energy intake, we adjusted the effect of BMI in an Analysis of covariance (ANCOVA) analysis. We used multiple logistic regressions for assessing the association between dietary quality indices and NAFLD in an adjusted model for sex, age, and BMI. Multiple logistic regressions adjusted for confounders was used for evaluating the relation between dietary quality indices and anthropometric and biochemical variables. The significance level was determined as P < 0.05. 

**Table 1.** Summary of Dietary Indices

	Description
**Dietary Energy density**	The method of Ledikwe et al. (24) was used to determine the ED of the diets. This method calculates ED from food only as energy (kcal)/weight of food (g) excluding nonenergetic beverages.
**Dietary diversity score**	This was calculated according to Kant et al. (28, 29) method. Five food groups based on the food groups introduced by the USDA`S Food Guide Pyramid (bread-grains, vegetables, fruits, meats and dairy) was considered. The main mentioned food groups were divided into 23 subgroups. These subgroups show the dietary diversity across the groups of the Food Guide Pyramid. For determining the consumers of each group we considered consumption of one-half of serving for each group. Each of the 5 food groups received a maximum diversity score of 2 of the 10 (5×2 = 10). Total score was the sum of the scores of each of the five main groups.
**Healthy Eating Index (HEI)**	The method which was introduced by Kennedy et al. (30) was used for calculating HEI. This index contains 10 different components. The amount of five groups of grains, vegetables, fruits, milk and meat groups as expressed in servings/day was considered as the first five components of HEI. 10 points were given to the intakes at or recommended amounts for each food group (30). If anybody consumed no servings of a food group received a score of 0. Between 0 and 10, the scores were calculated proportionately. The percent consumed of total fat and saturated fatty acids, were the 6^th^ and 7^th^ scores, respectively. The score of cholesterol intake and dietary variety was considered as the 8^th^ and 9^th^ components. A full score of 10 points was awarded for diets with < 30% energy from fat, < 10% energy from saturated fat and < 300 mg cholesterol. The number 10 was related to sodium. A full score of 10 was awarded to those not adding the table salt according to their report.
**Mean adequacy ratio (MAR)**	The ratio of daily individual intakes to standard recommended amounts for subject’s sex and age category was used for calculating nutrient adequacy ratio (NAR). The standard recommended amounts were based on the RDA (Recommended Dietary Allowances) and the DRI (Dietary Reference intake) (33). The NAR for ten nutrients including vitamin D, vitamin B12, vitamin B6, vitamin A, vitamin B2, niacin, vitamin C, calcium, iron, and zinc was estimated according to the mentioned formula. Mean adequacy ratio (MAR) was calculated as the sum of NARs divided on the number of nutrients (n = 10)

## 4. Results

Anthropometric and biochemical indices among case (non – alcoholic fatty liver patients) and control groups are shown in Table 2. Patients with NAFLD had higher weight, body mass index and waist circumference compared to the control group. Patients with NAFLD had higher level of liver enzymes, triglyceride, LDL, total cholesterol, albumin, blood urea nitrogen, and uric acid. Table 3 shows the dietary intakes of patients with NAFLD and healthy ones. Patients had higher intake of total calorie which remained higher even after adjustment for BMI. Although carbohydrates and fat intake was higher among patients, they consumed lower amounts of antioxidant micronutrients such as vitamin C, vitamin A, selenium, and vitamin E. They had lower intake of calcium and vitamin D as well. There was no case of under or over reporting who reported a total daily energy intake outside the range of 800–4200 kcal in this population. Table 4 shows the dietary quality indices among patients with NAFLD and the control group. Patients had lower values of healthy eating index, dietary diversity score, mean adequacy ratio of nutrients intake as well as nutrient adequacy ratio mostly regarding antioxidants, calcium and vitamin D. However, they had higher amount of energy density. Table 5 shows the association between dietary quality indices and NAFLD. The results showed that all the dietary indices were associated with NAFLD in a marginally significant level. Table 6 presents the association between HEI and anthropometric and biochemical indices. Overweight and obesity and high LDL level were significantly correlated with HEI. 

**Table 2.** Anthropometric and Biochemical Indices Among the Case (Non–Alcoholic Fatty Liver Patients) and Control Groups

Variables	Groups, Mean ± SD		P value
	Case (n = 100)	Control (n = 100)	
**Men, No.**	38	42	-
**Age, y**	37.9 ± 6.9	37.9 ± 6.9	0.09
**Anthropometric Variables**			
**Weight, kg**	55.9 ± 15.6	73.2 ± 12.9	0.04
**Body Mass Index, kg/m^3^**	27.9 ± 3.1	25.0 ± 2.0	0.03
**Waist, Men**	109 ± 13.2	102 ± 12.5	0.05
**Waist, Women**	96.1 ± 9.9	95.9 ± 9.1	0.04
**Blood pressure**			
**Systolic, mmHg**	129 ± 11.2	110 ± 10.1	0.08
**Diastolic, mmHg**	89.7 ± 4.5	77.6 ± 3.9	0.03
**Biochemical Variables**			
**White Blood Cell**	6695.1 ± 2432.8	6593.2 ± 2311.1	0.19
**Hemoglobin**	15.2 ± 3.4	14.5 ± 1.8	0.28
**Fasting blood sugar, mg/dl**	98.2 ± 7.6	80.1 ± 6.7	0.07
**Triglyceride, mg/dl**	218.2 ± 14.3	133.2 ± 12.2	0.01
**LDL, mg/dl**	129.9 ± 23.4	98.3 ± 13.0	0.01
**HDL, mg/dl**	48.4 ± 15.1	50.9 ± 17.3	0.41
**Tchol, mg/dl**	222.5 ± 42.1	179.8 ± 40.0	0.05
**AST **	53.0 ± 40.0	24.5 ± 12.4	0.01
**ALT**	74.9 ± 52.0	26.4 ± 13.0	0.01
**ALP^a^**	204.8 ± 94.0	183.0 ± 87.1	0.04
**Albumin**	4.8 ± 0.8	4.0 ± 0.5	0.05
**BUN^a^**	24.4 ± 4.5	11.8 ± 5.0	0.01
**Uric Acid**	6.7 ± 1.6	3.2 ± 1.0	0.01
**Creatinine**	0.9 ± 0.2	0.8 ± 0.2	0.23

^a^Abbreviations: ALP, Alkalinphosphatase; BUN, blood urea nitrogen

**Table 3.** Dietary Intake Among the Non Alcoholic Fatty Liver Patient, and Control Groups

Dietary intake	Groups, Mean ± SD		P value
	Case (n = 100)	Control (n = 100)	
**Total energy intake kcal**	2539 ± 211^1^	2193 ± 202	0.03
**Total energy intake adjusted for BMI, kcal**	2391 ± 207	2071 ± 193	0.04
**Macronutrients**			
**Carbohydrates, gr**	395 ± 38	327 ± 35	0.03
**Fat, gr**	81 ± 16	67 ± 15	0.04
**Protein, gr**	69 ± 17	65 ± 14	0.11
**Fiber, gr**	10.9 ± 4.6	16.1 ± 5.9	0.07
**Vitamin D, μg.d**	2.1 ± 1.9	3.1 ± 2.6	0.09
**Vitamin C, mg/d**	45.8 ± 21.0	59.3 ± 25.2	0.08
**Calcium, mg/d**	923.1 ± 511.1	1296.3 ± 523.3	0.06
**Zinc, mg/d**	232.5 ± 94.1	257.3 ± 96.3	0.09
**Magnesium, mg/d**	8.4 ± 3.0	9.6 ± 4.6	0.06
**Vitamin B_6_, mg/d**	2.3 ± 1.1	2.2 ± 1.2	0.71
**Selenium, μg/d**	10.1 ± 7.6	16.3 ± 9.6	0.05
**Vitamin E, Iu/d**	1.59 ± 1.4	2.26 ± 1.7	0.02
**Vitamin A, IU/d**	571.3 ± 351.3	776.6 ± 361.2	0.05
**Vitamin B_2_, Iu/d**	2.1 ± 1.3	2.0 ± 1.2	0.053
**Iron, mg/d**	9.4 ± 3.7	9.1 ± 3.3	0.73
**Whole grains, g/d**	31.3 ± 69.2	61.0 ± 101.3	0.001
**Refined grains, g/d**	378.1 ± 189.1	267.1 ± 181.2	0.001
**Fruit, g/d**	297.3 ± 111.3	313.4 ± 197.3	0.19
**Vegetables, g/d**	293.7 ± 111.3	359.9 ± 251.1	0.01
**Nuts, g/d**	10.3 ± 9.1	9.1 ± 8.7	0.10
**Red meat, g/d**	84.9 ± 31.3	76.9 ± 30.1	0.11
**Fish and Poultry, g/d**	43.1 ± 26.3	40.2 ± 24.4	0.16
**Dairy, g/d**	331.1 ± 211.6	311.9 ± 203.6	0.23

**Table 4.** Dietary Quality Indices Among the Non-Alcoholic Fatty Liver Patient, and Control Groups

Dietary quality indices	Groups		P value^a^
	Case (n = 100)	Control (n = 100)	
**HEI^b^**	53.3 ± 17.1	63.9 ± 17.9	0.01
**DDS^b^**	5.9 ± 2.3	7.7 ± 2.6	0.06
**DED^b^**	1.7 ± 0.5	1.1 ± 0.5	0.05
**MAR^b^**	10.6 ± 4.1	14.1 ± 4.9	0.03
**NAR^b^of different nutrients**			
**Zinc**	0.9 ± 0.2	1.3 ± 0.4	0.03
**Iron**	0.7 ± 0.3	0.8 ± 0.3	0.81
**Calcium**	0.9 ± 0.4	1.2 ± 0.4	0.03
**Vitamin C**	1.6 ± 0.9	2.3 ± 1.1	0.03
**Vitamin D**	0.3 ± 0.1	0.6 ± 0.2	0.03
**Vitamin B_2_**	1.6 ± 0.6	2.3 ± 0.8	0.02
**Vitamin E**	1.1 ± 0.6	2.2 ± 1.0	0.01
**Vitamin A**	1.2 ± 1.0	1.9 ± 1.1	0.04
**Selenium**	0.9 ± 0.6	1.7 ± 0.7	0.03
**Vitamin B_6_**	1.2 ± 0.5	1.3 ± 0.6	0.54

^a^ P values are resulted from student t-test

^b^ Abbreviations: DDS, Dietary Diversity Score; DED, Dietary Energy Density; HEI, Healthy Eating Index; MAR, Mean Adequacy Ratio; NAR, Nutrient Adequacy Ratio

**Table 5.** Odds Ratio for Having NAFLD in Different Quartiles of Dietary Quality Indices

	Quartiles of dietary quality indices				P trend
	1	2	3	4	
**NAFLD in quartiles of HEI**	1.88^1^(0.76-2.95)	1.54 (0.64-2.67)	1.31 (0.56-2.56)	1^a^	0.07
**NAFLD in quartiles of DDS**	1.76 (0.68-2.89)	1.36 (0.59-2.33)	1.30 (0.56-2.14)	1^a^	0.09
**NAFLD in quartiles of ED**	0.53 (0.19-0.89)	0.71 (0.31-1.14)	0.77 (0.33-1.21)	1^a^	0.05
**NAFLD in quartiles of MAR**	2.03 (0.91-4.03)	1.74 (0.57-3.86)	1.36 (0.36-2.98)	1^a^	0.05

^a^Multiple regression model was adjusted for age, sex, and BMI

**Table 6.** Association Between Dietary Quality Indices (HEI), Anthropometric and Biochemical Indices

	Quartiles of HEI				P trend
	1	2	3	4	
**Overweight or obesity**	2.18^1^(0.86-3.25)	1.74 (0.61-2.17)	1.30 (0.46-2.36)	1^a^	0.03
**High LDL level**	2.76 (0.78-4.89)	2.26 (0.69-4.53)	1.68 (0.51-2.84)	1^a^	0.04
**High TG level**	2.53 (0.89-4.19)	1.71 (0.61-2.04)	1.27 (0.43-1.81)	1^a^	0.05
**High FBS**	2.13 (0.91-4.01)	1.71 (0.67-2.86)	1.32 (0.56-2.28)	1^a^	0.06

^a^ Multiple regression model was adjusted for age, and sex. In other models except for overweight and obesity BMI was also adjusted

## 5. Discussion

The results of the present study conducted among patients with NAFLD and healthy individuals showed that dietary quality indices might be associated with NAFLD. Furthermore, patients might consume lower amounts of antioxidants, calcium and vitamin D while they might have higher intake of calorie, carbohydrate and protein. Serum level of liver enzymes, lipid profiles and blood urea nitrogen might be also higher among patients. Patients with NAFLD might have higher body mass index and waist circumference.

Diet quality indices might be one of the most important factors in assessing the quality of a diet (40, 41). These indices assess the whole intakes of individuals, so, they can provide a view from whole dietary intakes of patients. Although there are some reports regarding the dietary intakes of patients with NAFLD, according to the best of our knowledge, there is no report on the quality of diet and whole diet indices among these patients. Previous studies among Iranian population showed that HEI could be a good indicator for evaluating dietary quality in this population (42, 43). Those subjects who have higher score of HEI, usually may intake higher fruit and vegetable and lower unsuitable fat. These might be related to lower rate of NAFLD to some extent. Evidence on dietary diversity score in this population also confirmed this index as a suitable score for evaluating dietary quality (44, 45). Dietary energy density is also inversely associated with dietary quality indices based on previous studies among Iranians (44, 46). Most previously published papers were conducted on healthy subjects. The results of the present study showed that HEI, MAR, NAR of calcium, vitamin D, and antioxidant vitamins might be lower in patients with NAFLD. Dietary intakes of patients might be somehow different compared to the healthy subjects. Patients might have higher intake of carbohydrate and fat. Previous studies also showed that simple carbohydrate and refined grains as well as fat were consumed higher by the patients (13-16). Even a review paper indicated that high fat diet could rapidly develop the NAFLD (10). Reviews in the field of nutrition and NAFLD have focused on the harmful effects of fats (14, 16). Although, there are some discrepancy regarding the role of carbohydrate or fat in the etiology of NAFLD (14, 15), the results of this study showed that both carbohydrate and fat might be responsible. The kinds of fat intake may also be important. In the present study patients with NAFLD might have higher intake of saturated fat and cholesterol. Previous studies also showed harmful effects from saturated fatty acids (14, 15). There was no difference between the two groups (case and control) regarding protein intake in the present study. The effects of protein intake on NAFLD have been poorly assessed. Studies are mainly limited to animal models in this regard. An increment in dietary protein content has been shown to reduce the risk of hepatic fat accumulation during a high fat diet both in human and rodents (47-50). However, protein malnutrition also may lead to steatosis (51-53). In the present study, antioxidant intakes such as vitamin C, vitamin E, and selenium might be lower in patients. Previous reports also confirmed lower antioxidant intake in patients with NAFLD (54). Even the serum level of vitamin C is lower in patients with NAFLD, and vitamin C and vitamin E have important roles in this disease (55). In the present study, patients with NAFLD might have lower levels of calcium and vitamin D intake. Furthermore, they were more obese and overweight. A large body of literature focused on the role of calcium and vitamin D in obesity and overweight (56). Therefore, calcium and vitamin D could have a role in preventing and treating NAFLD probably via obesity and overweight control. In the present research patients consumed lower amount of vegetable and whole grain compared to healthy ones. So, it seems that they had fewer adherences to the healthy life style recommendations. They had lower intake of fiber which may be associated with higher body mass index, lipid accumulation, and higher levels of serum lipids (57). Previous reports also indicated the role of fiber consumption in NAFLD (16, 57, 58). In this research patients had higher levels of serum lipid, liver enzymes and BUN. There are several reports regarding these abnormalities among patients with NAFLD worldwide (25, 26). Although, we had some evidence regarding the biochemical difference between patients with NAFLD and healthy subjects, it was important that we found the same results in the present study in Iran. The case-control design of the present study was a limitation of this study. The exposure data gathering was performed after identification of outcome. Therefore, exposure data may indicate the current exposure, neither past nor permanent. However, this is the nature of case control study. The current dietary intake of the patients might be affected by diagnosis of NAFLD. The dietary intake of such patients may change after diagnosis. Although, we tried to assess the real usual dietary intake of the patients but the case-control nature of this study might bring some limitations in this regard. We suggest doing more prospective studies in future to clarify these associations in a prospective model. However, conducting prospective studies are very expensive and the budget limitation is a big barrier in developing countries. Small sample of patients and healthy group was another limitation. However, even with such a small sample we got significant results regarding the dietary quality indices as well as biochemical ones.

We used carriers of HBV as the control group; however, controls were not evaluated for the presence of cirrhosis and hepatocellular carcinoma (HCC). Considering that the mean age of controls was 37.8 (years), the risk of cirrhosis and HCC might be high in this control population. So, this is another limitation of the current study. Furthermore, liver ultrasonography has limitations in the diagnosis of patients with NAFLD (59). However, we also checked the liver enzymes status in this study and also considered the ALT and AST level besides the results of the ultrasonography. As we were going to compare the case and control groups for all possible anthropometric and biochemical and nutritional status, we did not match two groups for weight or other possible different variables to see and compare the difference between the two groups. However, this may cause some limitations in interpretations of the results. This drawback also exists regarding gender, and in considering the results of the present study we should also take into these limitations.

In conclusion, by considering the limitations of the current case-control study, it seems that the dietary quality indices might be related with NAFLD. Based on the results of the current case-control study, patients had lower consumption of vegetable and whole grains. Their antioxidant vitamins intake, calcium and vitamin D might be also lower than recommended amount and lower than healthy subjects while they might consume higher amount of energy and carbohydrate as well as fat. Patients with NAFLD had higher serum lipid, liver enzymes and BUN. Although in considering these results we should keep in mind that these results are from a case control study and this design has a lot of limitations as were discussed above. So, the present study is still far from a precise conclusion because of the nature of the case-control studies. Future prospective studies are needed to confirm these results in a large scale.
